# Voices of Stroke Survivors With Dysphagia: A Roy Adaptation Model‐Based Qualitative Analysis

**DOI:** 10.1002/brb3.71540

**Published:** 2026-07-03

**Authors:** Abdullah Avcı, Fadime Cennet Çokgün

**Affiliations:** ^1^ Vocational School of Health Services, Anesthesia Program Toros University Mersin Turkey; ^2^ Department of Neurology Mersin University Hospital Mersin Turkey

**Keywords:** adaptation, dysphagia, neurobehavior, stroke

## Abstract

**Aim:**

To explore the neurobehavioral and psychosocial adaptation experiences of stroke survivors with dysphagia using the Roy Adaptation Model as an analytical framework.

**Methods:**

This qualitative study employed a deductive design guided by the Roy Adaptation Model. Nineteen adults with acute stroke and clinically confirmed dysphagia were recruited from a university hospital in Türkiye between July and October 2025. Data were collected through semi‐structured, face‐to‐face interviews conducted within the first week after stroke onset. Interviews were audio‐recorded, transcribed verbatim, and analyzed using deductive content analysis based on the physiological, self‐concept, role function, and interdependence adaptive modes.

**Results:**

Analysis revealed four main domains of adaptation challenges. Participants experienced physiological disruption characterized by impaired swallowing, saliva control, and repeated swallowing behaviors. Emotional responses included fear of choking, embarrassment, and uncertainty regarding recovery, reflecting disruption in self‐concept. Role function was affected by loss of independence in feeding and daily activities, leading to increased dependence on caregivers. Interdependence was shaped by both social withdrawal and perceived support from family members and healthcare professionals during hospitalization.

**Conclusion:**

Stroke‐related dysphagia is associated with multidimensional neurobehavioral and psychosocial adaptation difficulties beyond its physiological impact. Early recognition of these adaptive challenges and provision of structured support systems may facilitate coping processes during the acute phase of stroke recovery.

## Graphical abstract

This graphical abstract illustrates the experiences of acute stroke survivors with dysphagia through the four adaptive modes of the Roy Adaptation Model. Dysphagia affected physiological functioning, self‐concept, role performance, and social relationships, while family and healthcare professional support facilitated adaptation. These findings emphasize the importance of comprehensive nursing care that addresses both physical and psychosocial needs during acute stroke recovery.

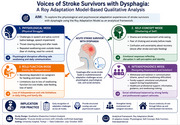

## 1 Introduction

Stroke remains a leading cause of death and long‐term disability worldwide (Feigin et al. 2022). Each year, more than 12 million people experience a stroke, and approximately 6.5 million deaths are attributed to stroke globally (Feigin et al. 2021).

In the post‐stroke period, various medical problems and complications may develop that negatively affect patients' quality of life and survival (Chen et al. 2023). One of the most common complications is dysphagia. The incidence and severity of post‐stroke dysphagia vary according to lesion location. Brainstem strokes are reported to be strongly associated with post‐stroke dysphagia, with incidence rates reaching up to 80% in some studies (Zhong et al. 2021). In addition, lesions involving deep brain structures and infratentorial regions (including the brainstem and cerebellum) have been associated with more severe swallowing impairment compared to supratentorial or cortical lesions (Kim et al. 2019). Prevalence rates reported in the literature range from 37% to 78%, and it is noted that the incidence of dysphagia is approximately 45% in the acute phase (Banda et al. 2022; Rofes et al. 2018; Song et al. 2024). Dysphagia occurs in 42%–67% of patients within the first three days after discharge (Arnold et al. 2016; Martino et al. 2015). Dysphagia that is not effectively managed can lead to serious complications such as malnutrition, dehydration, and aspiration pneumonia (Chang et al. 2022). Various studies in the literature have shown that dysphagia prolongs hospital stays, increases readmission rates, patient dependency, and healthcare costs (Arnold et al. 2016; Marin et al. 2020; Rofes et al. 2018). Additionally, it is known that dysphagia in individuals who have suffered a stroke leads not only to physical complications but also to psychological problems such as depression and anxiety (Horn et al. 2022). Complications arising from dysphagia also lead to delays in the rehabilitation process, negatively impact quality of life, and increase treatment and care costs (Bakhtiyari et al. 2020; Muehlemann et al. 2019). Therefore, effective management of post‐stroke dysphagia and supporting patients' adaptation to this condition is of vital importance in terms of prognosis.

Beyond its clinical complications, dysphagia also significantly complicates patients’ adaptation process, highlighting the need for structured nursing interventions. During this process, stroke management requires an interdisciplinary approach involving physicians, speech and language therapists, dietitians, rehabilitation specialists, and nurses. Among these professionals, nurses play a central role in supporting patients' physical and psychosocial adjustment as primary care providers (Madu and Ajibade 2025; Zhang and Qin 2024). Nursing models guide the systematic, holistic, and evidence‐based planning of care. Among these models, the Roy Adaptation Model (RAM) provides guidance for the systematic assessment of adaptation difficulties associated with dysphagia and for supporting the patient's adaptation to the new situation (Si 2024). The main reason for adopting RAM as the theoretical framework in this study is that the experience of dysphagia after stroke is a complex process that affects the individual's physiological, psychological, and social integrity in a multidimensional way. According to RAM, the individual is defined as an adaptive system in constant interaction with their environment, and health is associated with the individual's capacity to respond effectively to environmental stimuli (Roy 2011). Dysphagia developing after acute stroke causes impairments in patients' physiological functions (nutrition, respiration, etc.), while also bringing about changes requiring adaptation in psychosocial areas such as self‐concept, role functions, and the balance between dependence and independence (Li et al. 2022). In this context, RAM offers a comprehensive approach to identifying the stimuli experienced by patients, understanding their coping mechanisms, and evaluating their adaptive/maladaptive responses (Molu 2017). This model enables the systematic analysis of the experiences of stroke patients with dysphagia and facilitates the development of individualized intervention strategies in nursing care. For this reason, the study is structured based on RAM.

Previous qualitative studies have provided important insights into the challenges experienced by individuals with post‐stroke dysphagia and its impact on daily life (Helldén et al. 2018; Li et al. 2022). However, theoretical models that systematically explain adaptation processes in dysphagia remain limited. A comprehensive understanding of these experiences requires theoretically grounded approaches that address both physical and psychosocial dimensions. Theory‐based approaches to dysphagia management may enhance clinical care and support patients’ psychosocial adaptation processes (Awamura and Sakashita 2025). Therefore, a RAM‐guided approach has the potential to contribute to a more comprehensive and patient‐centered understanding of post‐stroke dysphagia.

The aim of this study was to explore the experiences of stroke survivors with dysphagia within the framework of the RAM. The research question was the following: How do stroke survivors with dysphagia describe their adaptation process across the four adaptive modes (physiological, self‐concept, role function, and interdependence) of the RAM?

## 2 Methods

### 2.1 Design

This study employed a qualitative research design. This study adhered to the Consolidated Criteria for Reporting Qualitative Research (COREQ) guidelines (Tong et al. 2007).

### 2.2 Setting and Participants

The population of this study consisted of patients who experienced acute stroke at a tertiary hospital located in southern Turkey. Participants were selected using criterion sampling, a type of purposeful sampling method. In qualitative research, sample size is not predetermined; data collection continues until data saturation is achieved, defined as the point at which no new codes or themes emerge (Saunders et al. 2018). Data saturation was reached after the 16th interview. Three additional interviews were conducted to confirm saturation, resulting in a final sample of 19 participants. Inclusion criteria were as follows: (1) being aged 18 years or older; (2) having experienced a first episode of acute ischemic or hemorrhagic stroke within 7 days of stroke onset; (3) having dysphagia confirmed by a neurologist through clinical bedside swallowing assessment; and (4) having no cognitive or perceptual impairment that would interfere with participation. Exclusion criteria included: (1) a previous history of ischemic or hemorrhagic stroke; (2) the presence of medical conditions other than stroke that could independently cause dysphagia (e.g., Parkinson's disease, multiple sclerosis, amyotrophic lateral sclerosis, head and neck cancer, or structural esophageal disorders), as determined through medical record review; (3) severe communication impairments (vision, hearing, or speech) preventing effective interviewing; and (4) a diagnosed psychiatric disorder.

### 2.3 Data Collection Tools

Research data was collected using two forms developed by reviewing the literature. The Information Form consists of seven questions designed to determine participants' sociodemographic and clinical characteristics, such as age, gender, stroke type, and stroke location. The semi‐structured interview guide consisted of core open‐ended questions aligned with the four adaptive modes of the Roy Adaptation Model. During interviews, example prompts and clarification questions were used when necessary to encourage elaboration and obtain richer descriptions. The interview guide is provided in Appendix 1.

**TABLE 1:** Sociodemographic and clinical characteristics of participants.

Participants	Age	Gender	Marital status	Education	Working status	Stroke type	Stroke duration (days)
1	62	Male	Married	Primary education	No	Ischemic	3
2	77	Female	Married	Literate	No	Ischemic	6
3	70	Female	Single	Primary education	No	Ischemic	4
4	63	Male	Married	Literate	Yes	Ischemic	3
5	55	Female	Married	University	Yes	Hemorrhagic	3
6	96	Female	Married	Primary education	No	Ischemic	5
7	80	Female	Married	Primary education	No	Ischemic	4
8	54	Male	Single	High school	Yes	Hemorrhagic	5
9	90	Female	Married	Primary education	No	Ischemic	3
10	68	Female	Married	Literate	No	Ischemic	5
11	90	Female	Married	Literate	No	Hemorrhagic	3
12	67	Male	Single	Primary education	No	Ischemic	3
13	60	Male	Married	High school	Yes	Ischemic	4
14	81	Male	Married	High school	No	Ischemic	4
15	51	Female	Single	Primary education	No	Ischemic	5
16	62	Male	Married	Literate	Yes	Ischemic	3
17	81	Male	Married	Primary education	No	Ischemic	4
18	76	Male	Married	Primary education	No	Ischemic	3
19	64	Female	Single	High school	No	Ischemic	5

### 2.4 Data Collection

Data were collected between July and October 2025 through face‐to‐face, in‐depth individual interviews conducted in a private room within the neurology clinic to ensure confidentiality. All interviews were conducted by the second researcher, a neurology clinic nurse with training in qualitative research methods. A semi‐structured interview guide was used. Prior to data collection, three pilot interviews were conducted to assess clarity and flow; minor revisions were made, and pilot data were excluded from analysis. Interviews lasted approximately 35 min and were audio‐recorded with participants’ permission. Field notes were taken to document nonverbal cues and contextual observations. In three interviews, a family member was present at the participant's request, either partially or throughout the session. Prior to the interview, participants were asked privately whether they preferred to be interviewed alone or with a family member present. The voluntary nature of participation was emphasized, and participants were informed that they were free to express their views openly. During these interviews, the researcher remained attentive to any signs of discomfort and ensured that responses reflected the participant's own perspectives. Each participant was assigned a unique code to maintain confidentiality.

### 2.5 Data Analysis

Data were analyzed using deductive content analysis as described by Elo and Kyngäs (2008), guided by the RAM. Audio recordings were transcribed verbatim and checked for accuracy by repeatedly listening to the recordings while reviewing the transcripts. Two researchers independently read all transcripts several times to gain familiarity with the data and to identify meaningful units. Initial codes were generated independently by each researcher. The researchers then compared their codes and discussed discrepancies until consensus was reached. This process ensured analytic consistency and enhanced dependability. Coding was conducted iteratively, moving back and forth between the raw data and the theoretical framework. As new insights emerged, earlier transcripts were revisited and re‐evaluated to ensure consistency in coding. The codes were grouped into subcategories based on conceptual similarity. These subcategories were then organized according to the four adaptive modes of the RAM (physiological, self‐concept, role function, and interdependence mode). An audit trail documenting coding decisions, category development, and theoretical alignment was maintained throughout the analysis process.

### 2.6 Rigor and Trustworthiness of the Study

Guba and Lincoln's (1989) criteria of credibility, transferability, dependability, and confirmability guided the study. Credibility was enhanced through prolonged engagement with participants, audio‐recording and verbatim transcription of interviews, and member checking to confirm the accuracy of findings. Interviews were conducted in Turkish and transcribed in the original language; selected quotations were translated into English and reviewed by a bilingual expert to ensure semantic equivalence. Dependability was supported by independent coding conducted by two researchers experienced in qualitative research. Coding was performed iteratively; both researchers initially coded the transcripts separately and then compared their coding line by line. Any discrepancies were discussed in detail, and codes were refined until full consensus was achieved. An audit trail documenting coding decisions and category development was maintained throughout the analysis process. Transferability was strengthened by including participants with diverse demographic and clinical characteristics and by providing detailed descriptions of the research process. Confirmability was ensured through data triangulation by comparing interview findings with clinical observations and medical records. All research materials, including transcripts, codes, and field notes, were securely stored.

## 3 Results

A total of 19 patients participated in the study. Participants' ages ranged from 51 to 96, with an average age of 70.89 ± 13.03. 52.6% of participants are female, 73.7% are married, and 47.4% are elementary school graduates. It was determined that 73.7% of participants were not working and 84.2% had suffered an ischemic stroke (Table 1). In this study, dysphagia experiences of patients who had an acute stroke were examined in four areas within the RAM framework. The findings are presented according to the main and sub‐themes identified for each area (Table 2). Overall, participants’ experiences were deeply embedded in daily hospital routines, particularly during assisted feeding, mealtimes, and interpersonal interactions, highlighting the pervasive impact of dysphagia on everyday functioning.

**TABLE 2:** Themes and subthemes according to Roy Adaptation Model.

Adaptation area	Theme	Subtheme	Sample statements from participant interviews
Physiological domain	Physical struggle	Challenges in speech and saliva control	“I try to speak, but my words don't come out properly. I can't control my saliva either… it just flows, especially when I'm in front of people.” (Participant 3)
		Throat clearing	“If I don't clear my throat, it feels like food is stuck there and I might choke. I have to do it all the time while eating.” (Participant 12)
		Repeated swallowing	“Even when I'm not eating, I keep swallowing… otherwise I feel like I will choke on my own saliva.” (Participant 2)
The concept of self	Shattering of self	Shame	“Food comes out of my mouth… people see it. I feel ashamed like a child. I don't want anyone to watch me eat.” (Participant 5)
		Fear	“Every bite feels like it could kill me. I think too much before swallowing… I'm afraid I won't survive it.” (Participant 2)
		Confusion and uncertainty	“Just three days ago, everything was normal, now I can't swallow. I have a tube in my nose, I'm being fed through it. I don't know when I'll get better or what tomorrow will bring.” (Participant 8)
Role function	Losing my independence	Becoming dependent	“I wait for my husband to feed me every spoon… I used to do everything myself. Now I depend on someone for eating and it hurts my pride.” (Participant 11)
		Limitations resulting from loss of function	“I can't even get a bite to eat anymore; I'm only feeding through the hole in my stomach. I feel like I am not eating at all.” (Participant 6)
Interdependence mode	Navigating dependence and isolation	Withdrawal and isolation in communication	“When saliva comes while I talk, I feel ashamed… so I stop talking to people.” (Participant 17)
		Family support	“My daughter stays with me all the time. She feeds me and I feel safe when she is here.” (Participant 9)
		Healthcare professional support	“The nurse told me how to take the food at the hospital and swallow it safely. When I follow her instructions, I feel less afraid.” (Participant 14)

### 3.1 Physiological Domain: Physical Struggle

Participants demonstrated limited physiological adaptation to dysphagia‐related stimuli in the acute phase of stroke, primarily reflected in compensatory behavioral responses such as repeated swallowing and frequent throat clearing. These behaviors indicated attempts to maintain airway safety and swallowing control despite disrupted physiological functioning. Participants described dysphagia as an ongoing bodily struggle that interfered with fundamental functions, including swallowing, speech, and secretion management.

### 3.2 Challenges in Speech and Saliva Control

Participants emphasized loss of oral control, particularly saliva leakage and speech impairment, which directly interfered with social presence and self‐confidence. These difficulties were particularly noticeable during face‐to‐face communication and mealtimes in shared hospital rooms, where participants reported increased self‐consciousness.

*“I try to speak, but my words don't come out properly. I can't control my saliva either… it just flows, especially when I'm in front of people.” (Participant 3)*

*“I don't even realize it, but my mouth stays open and saliva comes out. I feel like I've lost control of my face.” (Participant 18)*

### 3.3 Throat Clearing

Participants frequently feel the need to clear their throats during and after meals, as they feel saliva or food residue accumulating in their throats. This situation causes them to lose control while swallowing and also leads to longer mealtimes. Throat clearing behavior has emerged as an important physiological adaptation mechanism in terms of patients' swallowing safety and comfort. Participants stated that these sensations were most distressing during eating, especially when meals were consumed under observation in the hospital setting.

*“If I don't clear my throat, it feels like food is stuck there and I might choke. I have to do it all the time while eating.” (Participant 12)*

*“Food doesn't go down like before… it stays here (points to throat). I keep trying until I feel it move.” (Participant 16)*

### 3.4 Repeated Swallowing

Some participants reported feeling the need to swallow frequently throughout the day, even outside of mealtimes. This repeated swallowing, difficulty controlling saliva, and fear of choking are manifestations of a physiological response. Repeated swallowing affects patients' physical comfort and makes eating and talking in social settings difficult. This constant need to swallow was reported as interfering not only with eating but also with speaking during daily interactions.

*“Even when I'm not eating, I keep swallowing… otherwise I feel like I will choke on my own saliva.” (Participant 2)*

*“It never stops. I swallow again and again just to feel safe.” (Participant 9)*

### 3.5 The Concept of Self: Shattering of Self

Emotional responses indicated predominantly ineffective psychological adaptation to dysphagia‐related stimuli, as participants struggled to cognitively and emotionally adjust to sudden changes in swallowing ability. This maladaptation was reflected in heightened fear of choking, feelings of shame during eating, and persistent uncertainty regarding recovery. Dysphagia disrupted self‐perception and identity continuity, as participants perceived loss of bodily control as a threat to self‐integrity. Three sub‐themes were identified: fear, shame, and confusion/uncertainty, each reflecting different dimensions of disrupted psychological coping.

### 3.6 Shame

Participants stated that they felt embarrassed to eat in front of others due to the loss of control they experienced during meals. Participants emphasized their anxiety about losing control of their saliva or food during meals and stated that observing the reactions of others made them even more tense.

*“Food comes out of my mouth… people see it. I feel ashamed like a child. I don't want anyone to watch me eat.” (Participant 5)*

*“Even the patient next to me and her husband were watching… I felt humiliated. I just wanted to disappear.” (Participant 10)*

### 3.7 Fear

Participants emphasized that they experienced fear of choking while eating. This fear creates constant anxiety and unease not only while eating, but even as mealtimes approach. Participants stated that they thought long and hard before swallowing each bite, swallowed slowly, but still felt as if they were choking. This situation seriously undermines feelings of security during meals, increases psychological stress and anxiety, and makes eating in social settings nearly impossible. Some participants stated that they delayed mealtimes or preferred to eat alone due to fear.

*“Every bite feels like it could kill me. I think too much before swallowing… I'm afraid I won't survive it.” (Participant 2)*

*“Before every bite I stop for a moment and think about swallowing. I avoid talking during meals because I feel I might choke if I lose focus.” (Participant 4)*

### 3.8 Confusion and Uncertainty

Participants stated that the sudden onset of swallowing difficulties after stroke and the application of a nasogastric tube caught them unprepared. In particular, the insertion of the probe through the nose caused surprise and anxiety among participants, increasing both physical discomfort and awareness of the severity of the disease. This created noticeable anxiety in daily activities and reduced participation in social interactions during hospitalization. Participants experienced the shock of suddenly losing their ability to swallow while leading a normal life and having their nutrition provided through a tube; they felt helpless in the face of this unexpected change. This uncertainty was particularly evident during the transition to nasogastric feeding in the acute ward environment, which marked a sudden shift from independent oral intake to complete dependency.

*“Just three days ago, everything was normal, but now I can't swallow. I have a tube in my nose, and I'm being fed through it. I don't know when I'll get better or what tomorrow will bring.” (Participant 8)*

*“When they inserted the tube, I realized something serious happened. I don't understand how my life changed so fast.” (Participant 13)*

### 3.9 Role Function: Losing My Independence

Participants showed impaired adaptation in role performance, with increased dependence and reduced functional autonomy in daily living activities. This maladaptive response to dysphagia‐related functional limitations was particularly evident during feeding and self‐care activities, where participants increasingly relied on caregivers for basic needs. Participants stated that dysphagia was not only a physical impairment but also a disruption of previously independent social and family roles. Within the role function domain, the transition from independent functioning to caregiver‐supported living represented a major adaptive challenge, indicating difficulty in reorganizing role expectations in the acute phase of stroke. Two sub‐themes were identified: becoming dependent and limitations resulting from loss of function.

### 3.10 Becoming Dependent

Participants stated that dysphagia limited their ability to perform daily living activities and made them dependent on family members to meet their basic needs. This situation causes them to feel not only physically dependent, but also psychologically inadequate and vulnerable. Participants stated that they now experience a loss of morale due to constantly needing others to help them eat, something they used to do independently. This feeling of dependence reduces individuals' self‐confidence and negatively affects their ability to fulfill their social roles. This dependence was especially evident during assisted feeding routines, where participants relied entirely on caregivers for basic nutritional intake.

*“I wait for my husband to feed me every spoon… I used to do everything myself. Now I depend on someone for eating and it hurts my pride.” (Participant 11)*

*“Every meal I wait for someone to prepare the food and help me eat. I used to eat by myself without thinking, now even finishing a meal takes a long time.” (Participant 19)*

### 3.11 Limitations Resulting From Loss of Function

Participants indicated that dysphagia causes significant changes in eating habits along with the loss of swallowing function. Being unable to eat normally by mouth, being unable to taste food, and experiencing fear of choking while swallowing have meant a serious functional limitation for participants. The use of nasogastric tubes or percutaneous endoscopic gastrostomy in some participants has reinforced concerns that this limitation may be permanent. Not being able to take even a single bite by mouth and being fed through a hole in the abdominal area has caused individuals to lose their sense of control over their nutritional function. These limitations were most apparent during daily mealtime routines in the hospital, where oral intake was fully restricted or carefully supervised.

*“Nothing passes my lips anymore; I'm only feeding through the hole in my stomach. I feel like I am not eating at all.” (Participant 6)*

*“I don't even remember the taste of food. Everything is just fear now.” (Participant 3)*

### 3.12 Interdependence Mode: Navigating Dependence and Isolation

Social adaptation demonstrated a mixed pattern of both ineffective and partially effective responses to dysphagia‐related interpersonal stimuli. While participants experienced withdrawal from communication due to embarrassment, speech difficulties, and fear of social judgment, indicating maladaptive coping, they also relied heavily on external support systems as a compensatory adaptation strategy. Family members and healthcare professionals were identified as key sources of emotional reassurance and practical assistance, facilitating partial restoration of confidence in daily functioning. However, despite this supportive network, persistent feelings of loneliness and communication avoidance suggested that social adaptation remained limited during the acute phase of stroke.

### 3.13 Withdrawal and Isolation in Communication

Participants experienced withdrawal from communication and feelings of isolation due to swallowing difficulties and the constraints of the hospital environment. Difficulties experienced during conversation or meals have created feelings of embarrassment and shyness. This withdrawal was particularly evident during shared mealtimes and conversations in hospital wards, where participants avoided interaction due to fear of embarrassment.

*“When saliva comes while I talk, I feel ashamed… so I stop talking to people.” (Participant 17)*

*“During hospital meals I turn my face away from others and try to finish quickly because I feel uncomfortable when people see me struggle while eating.” (Participant 1)*

### 3.14 Family Support

Participants emphasized that the physical and emotional support provided by family members was critical to their adjustment process in the hospital. Family support has served as a source of confidence and morale, facilitating patients' adaptation to the hospital process.

*“My daughter stays with me all the time. She feeds me and I feel safe when she is here.” (Participant 9)*

*“Without my husband, I don't think I could handle this process. He gives me strength.” (Participant 7)*

### 3.15 Healthcare Professional Support

Participants stated that the information and guidance they received from nurses and other healthcare team members gave them confidence in coping with swallowing difficulties. This support helped patients feel more confident in managing swallowing difficulties. This support included bedside education, guidance on safe swallowing techniques, and practical assistance during meal times such as positioning and food consistency recommendations.

*“The nurse told me how to take food in the hospital and swallow it safely. When I follow her instructions, I feel less afraid.” (Participant 14)*

*“They explain everything step by step… it makes me feel like I can manage this situation.” (Participant 15)*

## 4 Discussion

In this study, the dysphagia experiences of patients in the acute phase of stroke were examined within the RAM, revealing challenges across the physiological, self‐concept, role function, and interdependence modes. Consistent with previous literature, participants described not only physical swallowing difficulties but also psychological and social disruptions (Li et al. 2022; Zhang et al. 2025). The sudden onset of stroke, combined with uncertainty and heightened anxiety, appeared to intensify both physiological vulnerability and psychosocial distress. Importantly, the severity and expression of dysphagia may also be influenced by clinical factors such as lesion localization and the time elapsed since stroke onset. For example, infratentorial lesions have been associated with higher rates of dysphagia compared to supratentorial strokes (Daniels et al. 2017), and experiences in the acute phase may differ from those reported in subacute or chronic stages as adaptation gradually occurs (Mao et al. 2024). Although these variables were not examined directly in this study, acknowledging their potential influence may help contextualize our findings and guide future research.

The physiological domain of RAM encompasses the responses individuals' bodies give to situations that threaten their health and the processes of adapting to these responses. The difficulties participants experienced with speech and saliva control, throat clearing, and repeated swallowing in the study clearly demonstrate the physiological effects of dysphagia. This finding has been similarly reported in the literature; for example, patients experiencing dysphagia after a stroke often have problems with speech and saliva control, throat clearing, and repeated swallowing (Hussain 2024; Li et al. 2022; Moloney and Walshe 2018). This situation negatively affects both safe swallowing capacity and daily communication, reducing patients' quality of life (Al Rjoob et al. 2022; Bakhtiyari et al. 2020). This finding is important because it shows that dysphagia after stroke is not merely a physiological problem, but also a multidimensional problem that directly affects patients' daily living activities and social interactions. In clinical practice, nurses and other healthcare professionals need to recognize changes in patients' swallowing and speech functions early on and plan individualized interventions. In this context, multidisciplinary nutrition management practices reported in the literature provide an important model; the comprehensive care provided by multidisciplinary teams significantly improves both swallowing function and nutritional status in patients experiencing dysphagia after stroke (Yan et al. 2022).

The self‐concept domain of RAM refers to individuals' perceptions of themselves, their self‐esteem, and their efforts to maintain identity integrity. The study found that participants experienced embarrassment, fear, and uncertainty due to dysphagia. The data obtained reveal that dysphagia after stroke has both physiological and psychological dimensions and constitutes a problem that particularly threatens self‐perception. Similarly, the literature indicates that individuals experiencing dysphagia withdraw from social settings and prefer solitude due to the loss of control they experience during meals, and that this situation leads to a decrease in self‐esteem (Li et al. 2022). Additionally, fear of drowning and embarrassment negatively affect patients' psychological adjustment and social participation (Bushuven et al. 2022; Kalkers et al. 2022). These findings reveal that post‐stroke dysphagia disrupts individuals' sense of identity, making psychosocial adjustment difficult. In clinical practice, nurses must be sensitive not only to physiological swallowing problems but also to changes in patients' self‐perception. In this regard, individual support, family involvement, and directive nursing interventions provided in the hospital setting can help patients maintain their sense of self‐integrity by strengthening their psychological adjustment. Future research should examine the long‐term effects of dysphagia on self‐perception and the effectiveness of nursing interventions aimed at reducing these effects.

The role function area of RAM refers to individuals' capacity to fulfill their social and family roles and the processes of adapting to these roles. The study found that participants' transition from their former independent roles to a position of receiving care, particularly the limitations that arose in eating and nutritional functions, created significant adjustment difficulties. The findings reveal that dysphagia has psychosocial dimensions beyond its physical effects and weakens the ability of individuals to maintain their role functions after acute stroke. The literature also reports that patients experiencing dysphagia after a stroke become dependent on family members for daily activities due to their limited independence, and that this situation negatively affects both their psychosocial adjustment and their quality of life (Karisik et al. 2025). In this context, nurses and other healthcare professionals in clinical practice should recognize difficulties in patients' daily living activities at an early stage and support their role functions through individualized interventions and rehabilitation plans. For example, providing supportive strategies and care plans for daily living activities in conjunction with swallowing therapy can help patients maintain their independence and continue performing their roles. This finding is supported in the literature; indeed, a prospective longitudinal study reported that swallowing function and rehabilitation motivation significantly predicted improvement in daily living activities among stroke patients (Tseng et al. 2024).

The reciprocal bond domain of RAM encompasses the processes by which individuals receive support through social relationships and maintain their social bonds. Participants in the study reported experiencing both social isolation and support due to dysphagia simultaneously. Social isolation has led individuals to withdraw from social settings due to feelings of loss of control and embarrassment experienced during meals. This situation has been similarly reported in the literature (Hussain 2024; Li et al. 2022). The findings indicate that dysphagia after stroke is a multifaceted problem that affects not only swallowing function but also social relationships and adaptive skills. At the same time, family support emerged as a central source of strength in participants’ adaptation processes. In the Turkish sociocultural context, where familial caregiving roles are often strongly emphasized, close family involvement may play a particularly prominent role in daily care and rehabilitation. Family members supported patients during meals, personal care, and therapeutic activities, thereby facilitating the maintenance of role function and psychosocial adjustment. Similarly, the information, guidance, and swallowing training provided by healthcare professionals has also strengthened patients' coping skills. The literature also reports that individuals experiencing dysphagia significantly improve their psychological adjustment and quality of life through the support they receive from social support systems (Patterson et al. 2013). Structured psychosocial interventions, including caregiver education programs and group‐based rehabilitation approaches, may further strengthen adaptive capacity by reducing isolation and enhancing coping resources (Budak and Yıldız 2025; Yang et al. 2023). Additionally, continuous nursing interventions incorporating follow‐up care, individualized education, and home‐based support may contribute to sustained adaptation and improved functional outcomes (Xu and Zhan 2025).

As this study included only patients in the acute phase (within 7 days post‐stroke), findings primarily reflect early‐stage adaptation to dysphagia. Within the RAM framework, dysphagia functioned as a focal stimulus, while hospitalization‐related conditions such as nasogastric feeding, dependence on caregivers, and environmental constraints acted as contextual stimuli influencing adaptive responses. Overall, adaptation was predominantly limited across self‐concept and role function domains, whereas interdependence‐related support contributed to partial adaptive stabilization. These findings suggest that early post‐stroke dysphagia represents a dynamic but constrained adaptation process. Therefore, structured, staged rehabilitation approaches delivered by interdisciplinary teams including nurses and speech‐language therapists are essential to enhance adaptive capacity across all RAM modes. Future studies should further examine how adaptive responses evolve across different phases of stroke recovery and how targeted interventions can optimize both physiological and psychosocial outcomes.

### 4.1 Relevance to Clinical Practice

The findings offer clinically relevant insights for nursing care structured according to the four domains of the RAM. In the physiological domain, systematic assessment of swallowing function, saliva management, and aspiration risk should be integrated into routine care. Structured precaution protocols and individualized guidance on posture, pacing, and food consistency may reduce complications and enhance patient comfort. Within the self‐concept domain, early identification of emotional distress related to dysphagia—such as anxiety, embarrassment, or reduced self‐confidence—is essential. Supportive communication, brief structured screening, and timely referral to mental health professionals may strengthen psychological adjustment. Regarding the role function domain, evaluating independence in activities of daily living and coordinating early interdisciplinary rehabilitation can support patients’ continuity of roles and autonomy. Integrating swallowing management with functional rehabilitation may reduce perceived dependence. In the interdependence domain, culturally sensitive, family‐inclusive approaches are particularly important. Structured family education and caregiver engagement can reinforce adaptive processes, while facilitating safe social interaction may mitigate withdrawal and isolation. In addition, continuous nursing approaches incorporating follow‐up communication, health education, home visits, and individualized rehabilitation planning may strengthen continuity of support and improve swallowing outcomes, psychological well‐being, functional recovery, and treatment adherence among patients with post‐stroke dysphagia (Xu and Zhan 2025). Aligning nursing interventions with these adaptive domains may promote more holistic management of post‐stroke dysphagia and support patients’ overall adaptive capacity during the acute phase.

### 4.2 Limitations

This study has some limitations. First, participants were selected only from a center located in the southern region of Turkey. This situation may limit the generalizability of the findings to stroke patients in different geographic regions. Secondly, only individuals who had suffered a stroke during the acute phase were included in the study. Therefore, the findings reflect early‐stage adaptation experiences related to dysphagia and may differ from the experiences of individuals in the subacute or chronic stages. Thirdly, interviews with participants experiencing communication difficulties sometimes made it challenging to focus deeply on the subject. Because participants struggled with saliva control or had difficulty producing words, most responses were quite short and limited. Additionally, in three interviews, a family member was present at the participant's request, either partially or throughout the session. While no substantial differences in themes or content were observed compared to interviews conducted privately, the presence of a relative may have influenced the directness of responses or the authenticity of participants’ expressions.

## 5 Conclusion

When interpreted within the RAM, this study indicates that acute stroke patients with dysphagia undergo complex and interrelated adaptation processes across physiological and psychosocial domains. Swallowing difficulties are closely linked with disruptions in role function, communication withdrawal, and emotional distress, reflecting the multidimensional nature of early post‐stroke adaptation. Family involvement and professional guidance emerged as central supportive resources that may facilitate coping and psychosocial adjustment, particularly within the Turkish sociocultural context, where familial caregiving plays a significant role. These findings highlight the importance of comprehensive, family‐inclusive care approaches that address both physiological management and psychosocial needs during the acute stage of stroke. Nursing care should incorporate systematic assessment of social engagement, mealtime experiences, and emotional responses related to dysphagia, alongside individualized swallowing education and culturally sensitive psychosocial support. Such integrated interventions may strengthen adaptive capacity and support early recovery.

## Author Contributions

**Abdullah Avcı**: conceptualization, methodology, formal analysis, writing – original draft, writing – review and editing, visualization, project administration, supervision. **Fadime Cennet Çokgün**: investigation, data curation, formal analysis, writing – review and editing.

## Funding

This research received no specific grant from any funding agency in the public, commercial, or not‐for‐profit sectors. Open access publication charges may be covered under the Wiley–TÜBİTAK ULAKBİM Open Access Agreement.

## Conflicts of Interest

The authors declare no conflict of interest.

## Ethics Statement

Ethical approval was obtained from the Mersin University Clinical Research Ethics Committee (Decision No: 2025/739; Date: June 25, 2025) prior to data collection. Institutional permission was also granted by the participating hospital. Before the interviews, participants were informed about the purpose of the study, the voluntary nature of participation, the confidentiality of their information, and their right to withdraw at any time without consequences. Written and verbal informed consent was obtained from all participants. All identifiable data were anonymized and replaced with unique participant codes (e.g., Participant 1, Participant 2).

## Data Availability

Data will be made available on reasonable request.

## Appendix 1

### Semi‐Structured Interview Guide Based on the Roy Adaptation Model

**Table:**

***Physiological mode*Core question** *Can you talk about the changes that occur in your body due to difficulty swallowing after a stroke?* **Example prompts**– How has swallowing difficulty affected your eating or drinking?– Have you experienced problems related to saliva control, coughing, choking, or speaking?– How have these physical changes affected your daily activities or comfort? ***SELF‐CONCEPT MODE*Core question** *How did difficulty swallowing affect you physically and emotionally after stroke??* **Example prompts**– How has dysphagia affected the way you see yourself?– Did you experience emotions such as fear, embarrassment, anxiety, or uncertainty?– How did swallowing problems influence your confidence or emotional well‐being? ***Role function mode*Core question** *How were your responsibilities to yourself affected when you had difficulty swallowing?* **Example prompts**– How did swallowing difficulty affect your independence in daily life?– Were there activities you could no longer perform on your own?– Did you feel dependent on others for eating, self‐care, or daily activities? ***Interdependence mode*Core question** *How were your relationships with people around you affected when you had difficulty swallowing?* **Example prompts**– How did swallowing difficulty affect your communication with family members, caregivers, or healthcare professionals?– Did you feel socially supported or isolated?– What kind of support was most helpful for you during this period?
